# Association of *CTRC* and *SPINK1* gene variants with recurrent hospitalizations for pancreatitis or acute abdominal pain in lipoprotein lipase deficiency

**DOI:** 10.3389/fgene.2014.00090

**Published:** 2014-04-22

**Authors:** Karine Tremblay, Camélia Dubois-Bouchard, Diane Brisson, Daniel Gaudet

**Affiliations:** ^1^Department of Medicine, Université de MontréalMontreal, Canada; ^2^ECOGENE-21 Clinical Research CenterSaguenay, QC, Canada

**Keywords:** severe hypertriglyceridemia, lipoprotein lipase deficiency (LPLD), pancreatitis, *CTRC* gene, *SPINK1* gene, *LPL* gene

## Abstract

**Background:** There are important inter-individual variations in the incidence and severity of acute pancreatitis in patients with severe hypertriglyceridemia. Several genes involved in triglyceride-rich lipoprotein metabolism or serine proteases pathways are known to influence the risk of pancreatitis.

**Aim:** To evaluate the association between genes regulating serine proteases, chymotrypsin C (*CTRC*) and serine peptidase inhibitor kazal type1 (*SPINK1*), and recurrence of hospitalizations for acute pancreatitis or severe abdominal pain in patients with Lipoprotein Lipase Deficiency (LPLD), a rare and extreme monogenic model of severe hypertriglyceridemia and pancreatitis.

**Method:** The *CTRC* and *SPINK1* genes promoter and coding regions sequencing has been performed in a sample of 38 LPLD adults (22 men and 16 women) and 100 controls (53 men and 47 women). Estimation of the association of *CTRC* and *SPINK1* gene variants or combinations of variants with history of hospitalizations for pancreatitis or acute abdominal pain in LPLD was investigated using non-parametric analyses with correction for multiple testing and logistic regression models controlling for age, gender, family history, and life habits.

**Results:** Gene sequencing followed by genotype-stratified analyses of the *CTRC* and *SPINK1* genes in LPLD and controls revealed a positive association between recurrence of hospitalizations and the rs545634 (*CTRC*)—rs11319 (*SPINK1*) combination [*OR* = 41.4 (*CI*: 2.0–848.0); *p* = 0.016]. In all models, a positive family history of pancreatitis was a significant predictor of recurrent hospitalizations independently of the contribution of *SPINK1* or *CTRC* (*p* < 0.001).

**Conclusion:** These results suggest that a positive family history of pancreatitis and genetic markers in the serine protease pathways could be associated with a risk of recurrent hospitalization for acute pancreatitis in severe hypertriglyceridemia due to LPLD.

## Introduction

Very severe hypertriglyceridemia (defined as fasting plasma TG concentration >10 mmol/L or 900 mg/dl) increases the risk of acute pancreatitis and has a prevalence of approximately 1/600 in North America (Johansen et al., 2011). Both severe hypertriglyceridemia and acute pancreatitis are associated with important clinical and socio-economic burden (Gaudet et al., 2013). However, there are important inter-individual variations in the incidence and severity of acute pancreatitis in patients with severe hypertriglyceridemia. Although rare, some extreme forms of severe hypertriglyceridemia and pancreatitis risk exist. This is the case of Lipoprotein Lipase Deficiency (LPLD) [MIM: 238600—Familial hyperchylomicronemia]. LPLD is a rare monogenic disease transmitted on an autosomal recessive mode (Monsalve et al., 1990; Ma et al., 1991; Mattei et al., 1993; Brunzell and Deeb, 2001). It is associated with recurrent, severe abdominal pain, increased risk of acute pancreatitis and other morbidities such as pulmonary embolism-like syndrome, coronary heart disease with or without atherosclerosis, and metabolic consequences of pancreatic insufficiency, including insulinopenic diabetes (Brunzell and Deeb, 2001; Tremblay et al., 2011). Although rare, LPLD is the most common form of the Familial Chylomicronemia Syndrome (FCS). The estimated worldwide prevalence of LPLD is 1–2:1,000,000 (Fredrickson et al., 1978). Numerous loss-of-function lipoprotein lipase gene (*LPL*) [MIM:609708] mutations were identified, but only a small number are null alleles, recognized to be responsible to the markedly reduced or absent lipoprotein lipase activity observed in LPLD (Brunzell and Deeb, 2001). LPLD results in chylomicrons accumulation and severe hypertriglyceridemia (>10 mmol/L) in the fasting state, and is associated with characteristic clinical signs such as eruptive xanthomas, lipaemia retinalis, and hepatosplenomegaly (Brunzell and Deeb, 2001). Other causes of FCS are documented, such as apolipoprotein C-II (*APOC2*), glycosylphosphatidylinositol-anchored high density lipoprotein-binding protein 1 (*GPIHBP1*), lipase maturation factor 1 (*LMF1*) or apolipoprotein A-V (*APOA5*) gene deficiencies, which all result in functional LPLD (Surendran et al., 2012). Currently, no available lipid-lowering drug therapy is effective to control the risk associated with LPLD, and affected patients typically do not respond to fibrates or niacin (Brisson et al., 2010; Zhou and Sahin-Toth, 2011; Beer et al., 2012). LPLD, like all other causes of FCS, is predominantly treated by severe dietary fat restriction and the use of medium-chain triglycerides (MCT) (Brunzell and Deeb, 2001), which does not fully eliminate the risk of pancreatitis or disease progression, while interfering with patient's quality of life on a daily basis. Recently, *LPL* gene replacement therapy (Gaudet et al., 2010, 2012) has been approved in Europe for severely affected LPLD patients. New emerging classes of treatments are in clinical development for FCS, including (among others) DGAT-1 inhibitors (Meyers et al., 2012), apoC3 antisense therapy, MTP inhibitors (Sacks et al., 2013) and peptide linker technologies.

As in many Mendelian diseases, important variations are noted in the clinical expression of LPLD. In particular, the incidence and the severity of abdominal pain and acute pancreatitis vary importantly across an individual's lifespan and between affected individuals, even in the same family. Although the pancreatitis risk is extremely high in LPLD (Tremblay et al., 2011), some patients do not experience a crisis before advanced age while others were frequently hospitalized since young age or eventually die from complications. Such heterogeneity in LPLD phenotypic expression complicates the individualization of risk evaluation and stratification.

The genetic basis of pancreatitis is now well documented and numerous candidate genes have been reported (Chen and Ferec, 2009; Whitcomb, 2010, 2013). All these genes could theoritically influence the trajectory of pancreatitis risk in LPLD. The variability of pancreatitis expression in LPLD is obviously complex and can also be affected by several environmental factors and by epigenetic regulatory mechanisms such as methylation, and/or microRNA (Jiang et al., 2004; Chahwan et al., 2011). In this study, we used a candidate gene approach to evaluate the association between the recurrence of hospitalization for acute pancreatitis or severe abdominal pain in LPLD and genes known to contribute to pancreatitis susceptibility. Specifically, we have selected the chymotrypsinogen C (*CTRC*) and serine protease inhibitor Kazal-type 1 (*SPINK1*) genes based on their important physiological role in serine proteases regulation and because they might have a functional relation with LPLD through the high density lipoprotein (HDL) particle which is significantly affected in this disease. In particular, the HDL cholesterol concentration, and the number of HDL particles are significantly decreased in LPLD. HDL is an efficient carrier of alpha(1)-antitrypsin and has been reported to play a significant role in proteases inhibition.

## Materials and methods

### Subjects

A total of 138 Caucasian adults have participated in this study: 100 normolipemic controls and 38 genetically confirmed patients with LPLD. All LPLD patients were identical-by-descent (IBD) homozygotes or compound heterozygous for null alleles in the LPL gene and presented fasting plasma TG values >10 mmol/L and clinical characteristics of FCS (Gaudet et al., 1998; Gagné and Gaudet, 2007; Tremblay et al., 2011). Null *LPL* alleles are associated with <5% normal LPL activity. Fasting plasma TG, cholesterol, non-esterified fatty acids, and apolipoprotein B (apo B) were measured as previously described (St-Pierre et al., 2001). Among the 38 LPLD participants, 18 had a history of recurrent (≥5) hospitalizations for severe abdominal pain or acute pancreatitis whereas 8 had not yet been hospitalized for such condition. Overall, approximately 80% of LPLD patients had already suffered from at least one episode of pancreatitis or abdominal pain requiring hospitalization and the number of hospitalizations per individual ranged between 0 and 96. Calculation of the number of hospitalizations for pancreatitis or severe abdominal pain was performed by questionnaire and by reviewing patient's medical charts. Applying the criteria of the Atlanta classification for pancreatitis (Banks et al., 2013), the hospitalizations having been considered in this study group all definite, probable and suspected pancreatitis episodes. Hospitalizations for conditions clearly not suspected to be related to FCS (eg., appendicectomy, parietal pain) were not considered in the analyses. The family history was considered “positive” when at least one known relative (available in our database) has already been hospitalized for acute pancreatitis. All LPLD patients have been followed in a Lipid Clinic, received nutritional counseling and were prescribed a strict type-1 adapted low fat diet. This study has been approved by the Local Ethics Review Board in accordance with the Declaration of Helsinki and all subjects gave informed consent.

### CTRC and SPINK1 sequencing

**Figure 1:**
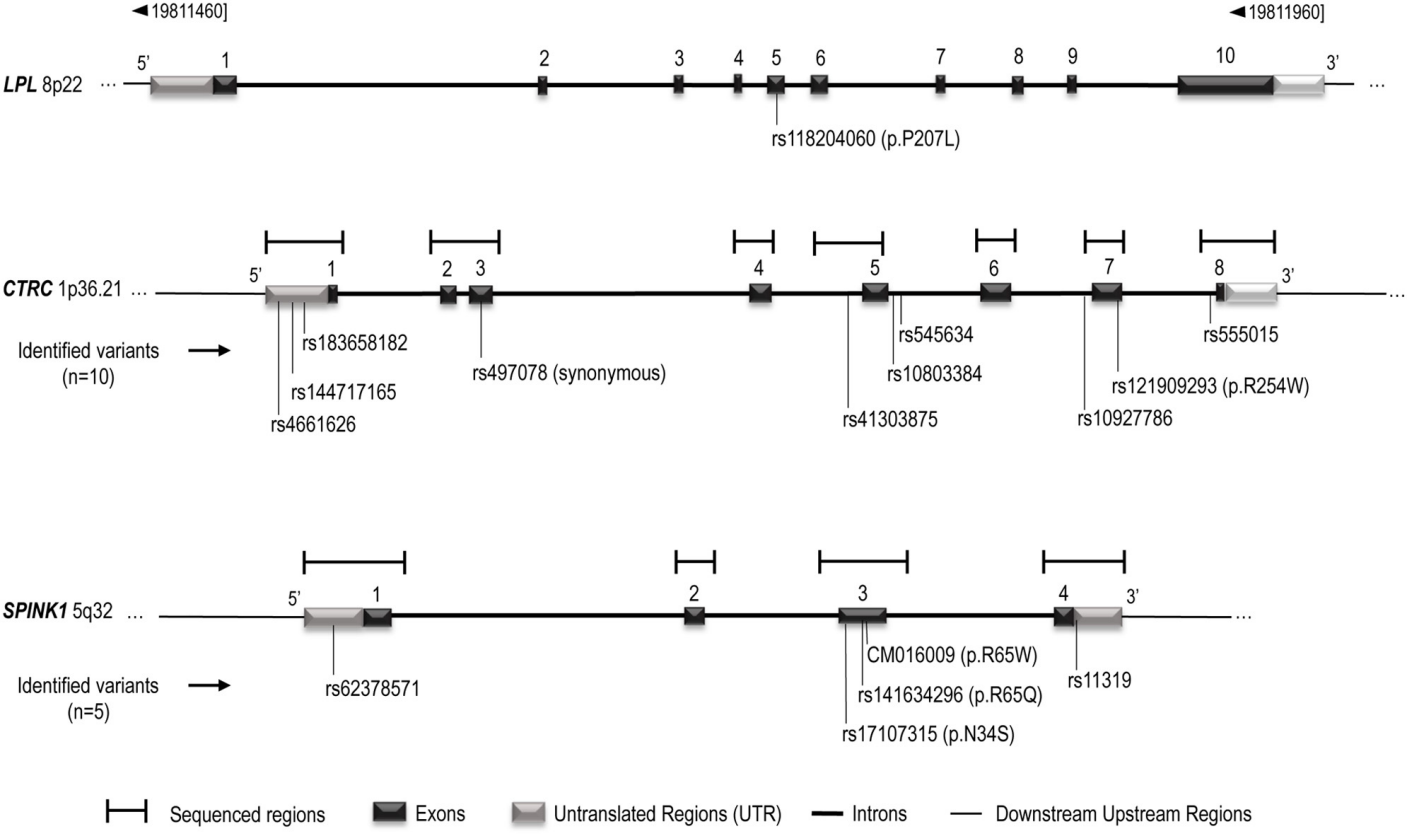
**Schematic representation of the *LPL*, *CTRC*, and *SPINK1* genes structure.** The gene representation is based on UCSC Genome Browser (http://genome.ucsc.edu/, February 2009, Santa Cruz, CA, USA) and Ensembl (http://uswest.ensembl.org/index.html, september 2013, Cambridge, United Kingdom), and is not scaled. *CTRC* and *SPINK1* sequenced regions and identified variants are indicated. Complementary informations on sequenced regions are avaible in Supplementary Table 1.

DNA has been extracted from blood leukocytes of all participants using Qiagen kit and following manufacturer's instruction (Qiagen Inc., Valencia, CA, USA). *CTRC* [1p36.21—GeneID: 11330] and *SPINK1* [5q32—GeneID: 6690] sequences information were obtained from UCSC Genome Browser (http://genome.ucsc.edu/, University of California, Santa Cruz, CA, USA). For *CTRC*, the PCR was divided into seven regions that spanned 3.1 kb, covering all eight coding and promoter sequences (Figure 1). For *SPINK1*, the PCR was divided into four regions that spanned 2.2 kb, covering all four coding and promoter regions (Figure 1). Analysis of sequences were performed with CodonCode Aligner (http://www.codoncode.com/aligner/, CodonCode Corporation Dedham, MA, USA). Oligonucleotide sequences as well as PCR amplification specific annealing temperatures are listed in the Supplementary Table 1. The PCR reactions were done in 25 μL volume containing 0–1 mM of Magnesium Chloride, 1X of Qiagen HotStart Taq PCR Buffer or 1X of NEB Taq PCR Buffer, 0.25 mM of dNTPs, 1 U/reaction of HotStart Taq DNA polymerase (Qiagen, Valencia, CA, USA or New England Biolabs Inc., Ipswich, Ma, USA), 0.2 μM of each primer and 20 ng/μl human genomic DNA. The PCR amplification was performed at 95°C for 10 min, 35 cycles of: first, 94°C during 30 s, secondly, annealing temperature for 30 s and thirdly, 72°C for 30 s. Finally, one cycle at 72°C for 7 min. Amplification products were purified with multiscreen PCR plates (MSNU 030 PCR, Millipore Corporation, Bedford, Ma, USA). Sequencing reactions were done in 10 μL volume containing 2 μL of purified and diluted PCR product, 0.3 μL of BigDye terminator v1.1 (Applied Biosystem Inc., Foster City, CA, USA), 1.75 μL of BigDye Terminator Sequencing Buffer 5X (Applied Biosystem Inc., Foster City, CA, USA) and 0.5 μL of the forward or reverse PCR primer (Supplementary Table 1). The sequencing reaction cycles were done following manufacturer's instructions (BigDye^®^ Terminator v1.1 Cycle Sequencing Kit). Reaction sequences were precipitated in 3 M of sodium acetate (Ambion^®^, Foster City, CA, USA) and 95% ethanol, resuspended in 10 μL of Hi-Di™ Formamide (Applied Biosystem Inc., Foster City, CA, USA) and analyzed on ABI PRISM 3100 Genetic Analyzer (Applied Biosystem Inc.).

### Statistical analyses

All statistical analyses were performed using SPSS software (IBM Corporation, SPSS Statistics for Windows, Versions 11.5.0, and 21.0, Armonk, NY, USA). Continuous variable comparisons between the different phenotypes were done using Kruskal-Wallis non-parametric tests combined to Mann-Whitney tests to perform inter-group comparisons. *P*-values are reported after Bonferroni correction for multiple testing. Chi-Square tests were used to compare dichotomic variables as well as minor allele frequencies and genotypes distribution between studied groups, and *z*-test was performed to inter-group comparisons. *P*-values were also adjusted using the Bonferroni method. For interaction analyses, significance level was fixed at *p* ≤ 0.005 since we have tested 10 SNPs combinations. Estimation of the association of *CTRC* and *SPINK1* variants or combinations with recurrent (≥5) hospitalizations for pancreatitis or acute abdominal pain in LPLD was also investigated using multinominal logistic regression models controlling for age, gender, and smoking status. Subjects with known alcohol abuse or known to be non-compliant to the diet were excluded from the study. In regression models, comparisons were made using normolipidemic controls as the reference. Results are reported for the groups of LPLD patients with ≥5 or <5 hospitalizations as odds ratio (OR) and 95% confidence interval (CI) at a significance level fixed at *p* < 0.05.

## Results

The characteristics of the two LPLD groups (defined on the basis of the number of hospitalizations for pancreatitis or abdominal pain) and controls are presented in Table 1. LPLD patients had significantly higher total triglyceride and cholesterol levels, and lower LDL-C and HDL-C than controls. Both LPLD groups were comparable for other lipid-lipoprotein variables, including total cholesterol, HDL-C, non-esterified fatty acids and total apolipoprotein B. LPLD subjects with recurrent hospitalizations were older, in addition to present a higher proportion of smokers than controls. They also presented a greater proportion of positive family history of pancreatitis than controls and LPLD subjects with <5 hospitalizations. All groups were comparable for body mass index.

**Table 1:** **Characteristics of the Participants**.

		**Controls**	**Severe hypertriglyceridemia (LPLD)**		***p*-value^f^**
			***<5 hospitalizations***	***≥5 hospitalizations***	
		***n* = 100**	***n* = 20**	***n* = 18**	
**DEMOGRAPHICS**					
Age [years; geometric mean (SD)]		41.8 (16.3)	43.2 (17.1)	53.2 (13.6)^2^	0.023
Gender [M/F (ratio)]		53/47 (1.1)	12/8 (1.5)	10/8 (1.3)	0.843
Body mass index [kg/m^2^; geometric mean (SD)]		24.0 (4.0)	22.7 (5.3)	22.6 (3.0)	0.303
Smoking status [n (%)]^a^		56 (56.0)	11 (68.8)	14 (93.3)^2^	0.020
Family history of pancreatitis [n (%)]^b^		6 (6.4)	7 (46.7)^1^	13 (92.9)^2,3^	<0.001
Hospitalizations [median (range)]^c^		0	1 (0–4)	16 (5–96)^3^	<0.001
**FASTING LIPID PROFILE**					
Total triglycerides [mmol/L; median (range)]		0.9 (0.3–1.8)	27.7 (14.9–60.8)^1^	33.3 (14.4–75.3)^2^	<0.001
Total cholesterol [mmol/L; median (range)]		4.2 (3.0–6.8)	6.9 (3.0–18.9)^1^	6.5 (3.6–15.1)^2^	<0.001
LDL-cholesterol [mmol/L; median (range)]		2.1 (1.2–3.5)	0.6 (0.2–2.3)^1^	0.5 (0.2–1.5)^2^	<0.001
HDL-cholesterol [mmol/L; median (range)]		1.3 (0.7–2.2)	0.4 (0.2–0.6)^1^	0.3 (0.2–0.5)^2^	<0.001
Plasma NEFA [mmol/L; median (range)]		0.5 (0.1–1.1)	0.5 (0.0–2.4)	0.5 (0.2–1.1)	0.629
Total apolipoprotein B [mmol/L; median (range)]		0.7 (0.4–1.4)	0.7 (0.3–1.8)	0.6 (0.3–2.1)	0.934
**SNP DISTRIBUTION^d^**					
*CTRC*	rs545634	13 (0.071)	1 (0.025)	5 (0.139)	0.158^g^
	rs10927786	27 (0.175)	10 (0.275)	9 (0.333)	0.055^g^
*SPINK1*	rs11319	6 (0.003)	1 (0.025)	4 (0.111)	0.063^g^
**SNP COMBINATION DISTRIBUTION^e^**					
*CTRC + SPINK1*	rs545634—rs11319	1 (1.0)	0	4 (22.2)^2,3^	<0.001^g^
	rs10927786—rs11319	1 (1.0)	0	0	0.847^g^

Abbreviations used: CTRC, Chymotrypsin C; F, Female; HDL, High-density lipoprotein; LDL, Low-density lipoprotein; LPLD, Lipoprotein lipase deficiency; M, Male; MAF, Minor allele frequency; NEFA, Non-esterified fatty acids; SD, Standard deviation; SNP, Single Nucleotide Polymorphism; SPINK1, Serine peptidase inhibitor, kazal type 1.

a Subjects who ever smoked (current or ex-smokers).

b At least one member of the family who had at least one pancreatitis episode. Complete family history was not available for all controls and LPLD subjects.

c Calculated on 13 subjects in ≥5 hospitalizations group.

d Presented as number of heterozygous and homozygous mutant subjects (MAF).

e Presented as number of subjects possessing at least one mutant allele in both identified variants (%) (meaning all subjects are heterozygous or heterozygous/mutant homozygous).

f Kruskal-Wallis (for continuous variables) and Pearson Chi-Square (for dichotomic variables). Group comparisons were done using Mann-Whitney or test-z adjusted with Bonferroni method. ^*1*^indicates significant difference (p ≤ 0.05) between <5 hospitalizations vs. controls; ^*2*^between ≥5 hospitalizations.vs. controls; and ^*3*^between <5 hospitalizations vs. ≥5 hospitalizations.

g Chi-square or Fischer exact tests p-value. Group comparisons were done using test-z adjusted with Bonferroni method. ^*1*^indicates significant difference (p ≤ 0.05) between <5 hospitalizations vs. controls; ^*2*^between ≥5 hospitalizations vs. controls; and ^*3*^between <5 hospitalizations.vs. ≥5 hospitalizations.

Table 1 also presents the compared frequency of the *CTRC* and *SPINK1* variants showing an association with recurrent hospitalizations for pancreatitis or acute abdominal pain while Table 2 presents the characteristics of all *CTRC* and *SPINK1* variants (*n* = 15) identified through gene sequencing in the different groups. All identified variants are single nucleotide polymorphisms (SNP) located in coding, non-coding or untranslated regions (Table 2 and Figure 1). The Supplementary Table 2 presents the associations in more details. Minor allele frequency comparisons between the studied groups revealed a trend of association with recurrent pancreatitis for only three SNPs (*p*-values < 0.2) (Table 1). Of note, among the eight LPLD individuals who had never been hospitalized for pancreatitis, the rs545634 was not observed while three heterozygous were observed for rs10927786 and one heterozygous for rs11319. We then performed two-by-two interaction analyses among the associated SNPs (Table 1). At a significance level of 0.005, only one SNP combination, *CTRC* (rs545634) and *SPINK1* (rs11319), showed a positive association after correction for multiple testing (*p* < 0.001). Genotype-stratified analyses confirmed that all subjects carrying this combination were heterozygotes (*p* < 0.001). We have then evaluated the association of this *CTRC* (rs545634) and *SPINK1* (rs11319) combination with recurrent pancreatitis in logistic regression analysis, including age, gender and smoking status as covariates (Table 3). Results of multivariate analyses confirmed the association of the rs545634-rs11319 SNPs combination with an increased odd of recurrent hospitalizations for acute pancreatitis or severe abdominal pain (*OR* = 41.4, *p* = 0.016). A positive family history of pancreatitis was also a significant covariate of recurrent hospitalizations in all groups (*p* < 0.001). When the family history was included in the regression models, the contribution of *SPINK1*-*CTRC* combination to the risk of hospitalization for pancreatitis or abdominal pain remained significant (*p* = 0.032).

**Table 2:**
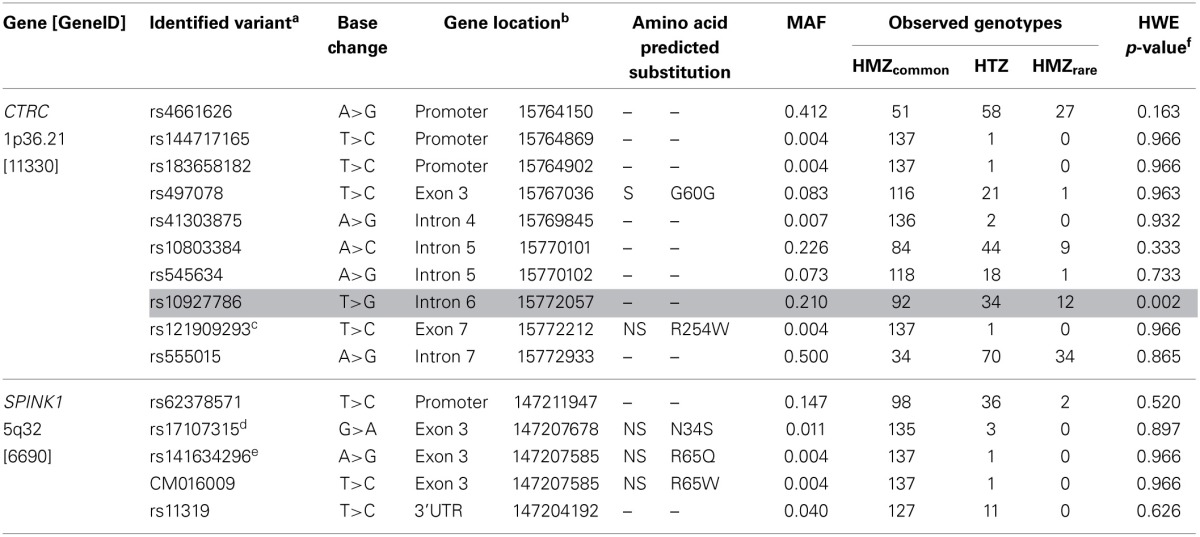
**Genetic Characteristics of the *CTRC* and *SPINK1* Variants Identified**.

Abbreviations used: MAF, Minor allele frequency; HMZ_common_, Homozygous for the common allele; HTZ, Heterozygous; HMZ_rare_, Homozygous for the rare mutant allele; HWE, Hardy-weinberg equilibrium; NS, non-synonymous; S, Synonymous; SNP, Single nucleotide polymorphism; UTR, Untranslated region.

^a^UCSC SNP reference number (http://genome.ucsc.edu/, February 2009).

^b^Position in UCSC Genome Browser (http://genome.ucsc.edu/, February 2009).

^c^Variant reported to be associated with chronic pancreatitis. (Masson et al., 2008; Zhou and Sahin-Toth, 2011; Beer et al., 2012).

^d^Variant reported to be associated with chronic pancreatitis, (Witt et al., 2000; Chen and Ferec, 2009) and acute pancreatitis. (Sanchez-Ramirez et al., 2012).

^e^Variant reported to be associated with chronic pancreatitis. (Boulling et al., 2007; Kiraly et al., 2007).

^f^Chi-square test p-value. Variant highlighted in gray is not in HWE (p ≤ 0.001).

**Table 3:** **Association of *CTRC* and *SPINK1* SNPs Combination and Family History with Recurrence (≥5) of Hospitalizations for Pancreatitis in Severe Hypertriglyceridemia due to LPLD**.

**Models^a^**	**LPLD subjects**					
	***<5 hospitalizations***			***≥5 hospitalizations***		
	***OR***	**[95% *CI*]**	***p*-value**	***OR***	**[95% *CI*]**	***p*-value**
*CTRC-SPINK1* SNPs combination	NA	–	–	41.4	2.0–848.0	0.016
Family history of pancreatitis	19.6	4.3 – 90.1	<0.001	410.6	29.4–5733.8	<0.001
*CTRC-SPINK1* SNPs combination + family history of pancreatitis	NA	–	–	148.6	1.5–14537.5	0.032

Abbreviations used: CI, 95% confidence interval; CTRC, Chymotrypsin C; LPLD, Lipoprotein lipase deficiency; NA, not applicable (the test is not valid due to the absence of cases), OR, odd ratio; SPINK1, Serine peptidase inhibitor, kazal type 1.

a Multinominal regression models tested, including age, gender and smoking status as covariates. The reference category are the normolipemic control subjects (Table 1).

## Discussion

Both pancreatitis and hypertriglyceridemia are complex traits and inter-individual variations are regularly observed in the expression of pancreatitis in patients with severe hypertriglyceridemia. Such differences are noticed even in a homogeneous group of individuals affected by LPLD, a disease characterized by extreme plasma triglyceride values and high risk of pancreatitis (Brunzell and Deeb, 2001; Tremblay et al., 2011). LPLD is a very rare disease and we thus report data on one of the largest sample of LPLD patients having been ever published. In this sample, we observed important variations in term of pancreatitis morbidity (assessed by the recurrence of hospitalizations), despite the fact that all patients were carrying the same *LPL* gene defect. Indeed, approximately 80% of LPLD adults in this study had been hospitalized at least once for acute pancreatitis (range: 1–96) but 20% had never suffered from pancreatitis, even at age >50. The results presented herein suggest that genetic markers in the serine protease pathways could influence the risk of recurrent hospitalizations for acute pancreatitis or acute abdominal pain in LPLD.

Clinically, the diagnosis of acute pancreatitis requires two of the following criteria: abdominal pain suggestive of acute pancreatitis, serum amylase and/or lipase activity at least three times greater than the upper limit of normal, and characteristic findings of acute pancreatitis on abdominal ultrasonography or on contrast-enhanced computed tomography scanning or magnetic resonance imaging (Frossard et al., 2008; Banks et al., 2013). However, due to severe chylomicronemia, FCS is by nature associated with spurious estimation of several laboratory values and patients can present with pancreatitis and normal amylase or lipase values. Furthermore, imaging results were not available in all patients medical files. This might lead to underestimating the number of definite pancreatitis episodes in these patients. To overwhelm this issue, we considered all cases of hospitalizations for acute abdominal pain which were suggestive of pancreatitis. This includes all definite, probable and suspected cases of pancreatitis.

Family history is an important predictor of risk of several lipid-related diseases, including pancreatitis. In this study, LPLD patients with ≥5 hospitalizations for pancreatitis or severe abdominal pain presented at least twice the proportion of positive family history of pancreatitis than that observed in patients with less hospitalizations and controls. The family history is obviously not reflecting the genetic background only and takes into account shared life habits and the environemental background.

Although hereditary forms of pancreatitis exist (LaRusch et al., 2012), the majority of genes having been associated with pancreatitis explain the predisposition, rather than the cause, of acute pancreatitis. All FCS causing genes, including *LPL* are well-documented pancreatitis susceptibility genes. Pancreatitis is a complex disease and the mechanisms explaining the pancreatitis risk in LPLD are not clearly understood. One hypothesis is that large chylomicrons lodged in pancreatic capillaries expose them to pancreatic lipase, with the subsequent release of free fatty acids through the hydrolysis of chylomicron-associated triglycerides. Local high concentrations of free fatty acids are thought to damage pancreatic cells leading to pancreatitis (Ross et al., 2004, 2006; Rip et al., 2005, 2006; Gaudet et al., 2010). Another hypothesis is that phospholipids and oxidized phospholipids at the surface of buyant-chylomicrons once hydrolyzed by phospholipase serve as triggers for proteases. In all cases, if enough proteases become activated intracellularly, they can overwhelm the first line of defense (pancreatic secretory trypsin inhibitor) and resist backup defenses (proteolytic degradation). Activated cationic trypsin can then trigger the entire zymogen activation cascade (Frossard et al., 2008). It is possible that mutations in *CTRC* and *SPINK1* genes contribute to this process and further increase the pancreatitis risk conferred by LPLD by either increasing the level of activated proteases, blocking active site of trypsin or decreasing proteases degradation. Indeed, mutant variants in *CTRC* and *SPINK1* genes are known to contribute to pancreatitis susceptibility (Chen and Ferec, 2009; Whitcomb, 2010). *CTRC* encodes the protease chymotrypsin C, a protease produced in small quantities by the acinar cells and found in an inactive form in the zymogen granules (Szmola and Sahin-Toth, 2007), which degrades trypsin when trypsinogen is prematurely activated in the pancreas (Szmola and Sahin-Toth, 2010). Genetic variations in *CTRC* can predispose to chronic pancreatitis by diminishing its protective trypsin-degrading activity (Szmola and Sahin-Toth, 2007; Rosendahl et al., 2008; Whitcomb, 2010). *SPINK1* encodes pancreatic secretory trypsin inhibitor, which prevents trypsin-catalyzed premature activation of zymogens within the pancreas and the pancreatic duct (Chen and Ferec, 2009; Ohmuraya and Yamamura, 2011; Kume et al., 2012). Mutations in this gene have been associated with hereditary pancreatitis (Ohmuraya and Yamamura, 2011; Ohmuraya et al., 2012), and it is thought to function in the prevention of trypsin-catalyzed premature activation of zymogens within the pancreas. *CTRC* and *SPINK1* have pleiotropic functions but their role in serine proteases (trypsin and chymotrypsin) physiology is shared in common. This might explain why the effect of the combination of variants in these two genes on risk of pancreatitis in LPLD was more pronounced than single variants effect in the present study: *CTRC*-rs545634, located in the 5th intron, could be responsible of an alternate splicing site which would affect this enzyme's integrity and its inhibitory effect, combined to *SPINK1*-rs11319, located in the 5'UTR, that could affect mRNA stability and be responsible of a lesser production of this trypsin inhibitor, may be compatible with an increased pancreatitis risk. Further functional studies on these variants are needed to elucidate the role of their mutant allele in this hypothetic loss of inhibitory function responsible of their associated pancreatitis risk in LPLD.

Trypsin and chymotrypsin are two structurally similar serine proteases synthesized in the pancreas as inactive zymogen precursors (trypsinogen and chymotrypsinogen) secreted in the duodenum via the pancreatic duct and converted to the mature, active enzyme by proteolysis (Frossard et al., 2008; Whitcomb, 2010). They recognize different protein substrates. Trypsinogen and chymotrypsinogen also enter the bloodstream, where they can be detected in serum (Artigas et al., 1981). In blood, serine proteases of the trypsin-like family have long been recognized to be critical effectors of biological processes as diverse as blood coagulation, fibrinolysis, and immunity (Antalis et al., 2011). Serum trypsin levels have been shown to be significantly higher in acute pancreatitis patients than in controls (Artigas et al., 1981). Mass spectrometry and western blotting have shown that HDL are efficient carriers of alpha(1)-antitrypsin (Ortiz-Munoz et al., 2009). HDL-associated alpha(1)-antitrypsin appears to be able to inhibit trypsin activity, extracellular matrix degradation, cell detachment, and apoptosis induced by proteases in human vascular smooth muscle cells. The number of HDL particles and their cholesterol content is in general very low in presence of severe hypertriglyceridemia, a feature systematically observed in LPLD. It is possible that low HDL-associated alpha(1)-antitrypsin is a factor contributing to LPLD morbidity, including hepatomegaly and deleterious liver effects, in presence of mutant alleles in protease-regulating genes, such as *CTRC* and *SPINK1*. Clinically, serum serine proteases have been directly associated with triglycerides metabolism, including GPIHPB1-related factors and glycosylphosphatidylinositol linkage (Deeg and Bowen, 2002). Severe hypertriglyceridemia induced by PEG-L-Asparaginase in patients treated for acute lymphocytic leukemia can induced acute pancreatitis in patients undergoing chemotherapy (Konig and Malek, 2012). This has been associated with increased levels of serum trypsin and elastase 10 and 20 days after beginning L-asparaginase therapy (Shimizu et al., 1998). Alcohol abuse is associated with both hypertriglycridemia, and proteases activation (Joly et al., 1992). It is one of the most common cause of acute pancreatitis (Thrower et al., 2008). In this study, we excluded all known cases of alcohol abuse, although it is almost not viable long term in FCS due to nature of the disease and the huge risk of heavy alcohol consumption for these patients. The links between the proteases networks and lipid metabolism are complex and the connection with pancreatitis risk and other morbidities in LPLD remain to be documented.

This study has limitations. LPLD is an ultra-rare, and extreme cause of hypertriglyceridemia and is thus not representative of all causes of severe hypertriglyceridemia. The rarity of LPLD, implies that we have to deal with small sample size which also limits the power of association studies. Despite this, the results suggest that genetic markers in the protease cascade could be associated with the expression of pancreatitis in LPLD. However, functional analyses were not performed in this study and we cannot infer at this point that there is a physiological association between protease regulators and the recurrence of pancreatitis in presence of severe hypertriglyceridemia. Our findings need to be validated and replicated in larger LPLD and severe hypertriglyceridemia cohorts and the functional effect of identified variants needs to be characterized before this SNP-combination could be considered as a clinical biomarker of recurrence of pancreatitis in LPLD. Since other genes are known to confer pancreatitis susceptibility, additional analyses are needed to understand the complex genetic architecture of pancreatitis in LPLD. Such studies, including whole genome sequencing, resequencing of extremes, functional gene expression analyses, physiologicial and metabolic studies, are actually underway.

## Author contributions

Karine Tremblay co-designed the study, co-supervised the *CTRC* and *SPINK1* genes sequencing, performed the statistical analyses and drafted the manuscript. Camélia Dubois-Bouchard participated in the review of the literature, the selection of candidate genes and in *CTRC* and *SPINK1* genes sequencing activities under Karine Tremblay supervision. She also participated in the statistical analyses and to the writing of the manuscript. Diane Brisson supervised *LPL* genotyping, participated in statistical analyses and revised the manuscript. Daniel Gaudet co-designed and supervised the study, coordinated the clinical tasks, revised the manuscript and approved the version to be published. During the study, Camélia Dubois-Bouchard was a Université de Montréal M Sc. student and Karine Tremblay a Université de Montréal postdoctoral fellow, both under Daniel Gaudet supervision.

### Conflict of interest statement

The authors declare that the research was conducted in the absence of any commercial or financial relationships that could be construed as a potential conflict of interest.
