# Supplementary material for: Association of CTRC and SPINK1 gene variants with recurrent hospitalizations for pancreatitis or acute abdominal pain in lipoprotein lipase deficiency
Source: Front Genet. 2014 Apr 22;5:90. doi: 10.3389/fgene.2014.00090 (PMC4000989; doi:10.3389/fgene.2014.00090)
Supplement: Supplementary file 1 [file DataSheet1.DOCX]

***Supplementary Material***

**Association of CTRC and SPINK1 Gene Variants with Recurrent Hospitalizations for Pancreatitis or Acute Abdominal Pain in Lipoprotein Lipase Deficiency**

Karine Tremblay, PhD^1†^, Camélia Dubois-Bouchard, BSc^1†^, Diane Brisson, PhD, CCRP^1^, Daniel Gaudet, MD, PhD^1*^

^1^ Department of Medicine, Université de Montréal and ECOGENE-21 Clinical Research Center, Saguenay, QC, Canada.

^†^ Authors contributed equally.

This supplementary material has been provided by the authors to give readers additional information about their work.

Includes :

**Supplementary Table 1:** *CTRC* and *SPINK1* Sequencing Oligonucleotides and PCR Conditions

**Supplementary Table 2:** *CTRC* and *SPINK1* Mutant Genotypes Distribution Among Subject Studied Groups

**Reference**

**Supplementary Table 1**

*CTRC* and *SPINK1* Sequencing Oligonucleotides and PCR Conditions

| **Genes [GeneID]** | **Regions** | **Chromosome Location** ^a^ | **Primer Sequences ^b^** | **PCR Product Size (bp)** | **PCR Tm**  **(°C)** | **Sequenced Strand** |
| --- | --- | --- | --- | --- | --- | --- |
| *CTRC*  1p36.21  [11330] | Promoter + Exon 1 | 15764135-15765024 | F: 5’-ACCGCGTCTATTTCATGTTGTT-3’  R: 5’-ACTCCCCATCCCGTCCTT-3’ | 987 | 56 | F and R |
|  | Exon 2 + Exon 3 | 15766644-15767095 | F: 5’-AACAAGGCCTAGAGACCTGG-3’  R: 5’-TGCACAACTGAGTTACTGGG-3’ | 596 | 66 | R |
|  | Exon 4 | 15768818-15769071 | F: 5’-GGAAAGGACAATGGGAACAC-3’  R: 5’-GTCATTTGCTGGCTTTCCA-3’ | 384 | 56 ^c^ | R |
|  | Exon 5 | 15769796-15770169 | F: 5’-GTTTGTGAAGGACCCCTGAG-3’  R: 5’-TGTCTGTCACATGGTATGTGCT-3’ | 420 | 51 | F |
|  | Exon 6 | 15770972-15771318 | F: 5’-GTGGTCCGCACACTGTCTC-3’  R: 5’-GCTTACCCTGAGCCTGCTG-3’ | 416 | 61.7 | R |
|  | Exon 7 | 15772057-15772375 | F: 5’-GAGGCCAAATCTGTCCACTAA-3’  R: 5’-ATGCATGAATGAGTGAATAAATGA-3’ | 405 | 61.7 | F |
|  | Exon 8 | 15772888-15773371 | F: 5’-TGAGAGTAGGGGAACAGAGGG-3’  R: 5’-CCTGTTCGCAGCTTGTGAGA-3’ | 626 | 66 | F |
| *SPINK1*  5q32  [6690] | Exon 1 | 147212340-147211390 | F: 5’-CAGGCTTGAGAAGGGAATGA-3’  R: 5’-AACAAAGGGTCAGCCACATC-3’ | 641 | 56 | F and R |
|  | Exon 2 | 147211350- 147211062 | F: 5’-AAGGGTGGGGAATGAAAGAG-3’  R: 5’-ATATACGCCTGTGGGTTGGA-3’ | 334 | 56 | R |
|  | Exon 3 | 147209295-147209053 | F: 5’-GCAGAGGCATGACTTAAAACAA-3’  R: 5’-CATGCTAAGGTTTCAGTTGCTG-3’ | 644 | 56 | R |
|  | Exon 4 | 147207836-147207337 | F: 5’-AAGATTATAAATCTCAAACCTCTCCAA-3’  R: 5’-ATGAGGGAAACCCTGTCTGA-3’ | 387 | 51 | F |
|  | Promoter | 147204383-147204162 | F: 5’-TGGCAGAAGGCACAGACTC-3’  R: 5’-TGAAACATGCAAGGCAAAGA-3’ | 968 | 56 | R |

Abbreviations used: F = Forward primer, R = Reverse primer, Tm = Annealing temperature.

^a^ Position in UCSC Genome Browser (http://genome.ucsc.edu/, February 2009).

^b^ As obtained by Primer 3.([Untergasser, Cutcutache et al. 2012](#_ENREF_1))

^c^ Taq DNA polymerase Qiagen (Qiagen Inc., Toronto, Canada). Taq DNA polymerase New England Biolabs (New England Biolabs Inc., Ipswich, MA, USA) for all other PCR conditions.

**Supplementary Table 2**

*CTRC* and *SPINK1* Mutant Genotypes Distribution Among Subject Studied Groups

|  |  | **Controls** | **LPLD** | |  |
| --- | --- | --- | --- | --- | --- |
|  |  |  | *<5 hospitalizations* | *≥ 5 hospitalizations* |  |
| **Gene** | **SNP** ^a^ | n = 100 | n = 20 | n = 18 | p*-*value ^b^ |
|  |  | **HMZ** | | |  |
| ***CTRC*** | rs545634 | 1 (1.0) | 0 | 0 | NS |
|  | rs10927786 | 8 (8.0) | 1 (5.0) | 3 (16.6) | NS |
|  |  | **HTZ** | | |  |
| ***CTRC*** | rs545634 | 12 (12.0) | 1 (5.0) | 5 (27.8) | 0.101 |
|  | rs10927786 | 19 (19.0) | 9 (45.0) | 6 (33.3) | 0.028 |
| ***SPINK1*** | rs11319 | 6 (6.0) | 1 (5.0) | 4 (22.2) | 0.056 |

Abbreviations used: *CTRC* = Chymotrypsin C, HMZ = Homozygous, HTZ = Heterozygous, LPLD = Lipoprotein lipase deficiency, n = Number, NS= Non significant, SNP = Single Nucleotide Polymorphism, *SPINK1* = Serine peptidase inhibitor, kazal type 1.

^a^ Presented as number of HTZ or HMZ subjects for each identified variants (%).

^b^ Chi-square p-values compared to subjects who are homozygous for the common allele, test-z was made to compare columns proportion and adjusted with Bonferroni method. NS = Non significant at a 0.2 threshold.

**Reference**

Untergasser, A., I. Cutcutache, T. Koressaar, J. Ye, B. C. Faircloth, M. Remm and S. G. Rozen (2012). "Primer3--new capabilities and interfaces." *Nucleic Acids Res* **40**(15): e115.
